# Knowledge, attitude, and practice of depression among university students

**DOI:** 10.1002/brb3.70030

**Published:** 2024-09-18

**Authors:** Xuechao Wang, Cuiluan Li

**Affiliations:** ^1^ Department of Human Resources and Organizational Behavior Shandong University of Finance and Economics Jinan China; ^2^ Shandong Mental Health Center Jinan China

**Keywords:** attitude, depression, knowledge, practice, university student

## Abstract

**Introduction:**

This study aimed to investigate the knowledge, attitude, and practice (KAP) of depression among university students.

**Methods:**

A cross‐sectional survey was carried out across randomly selected universities in Shandong Province from October 25, 2023, to November 8, 2023. Demographic information and KAP scores were assessed through the administration of questionnaires. The reliability of the questionnaire was confirmed with a Cronbach's alpha coefficient of 0.816 and the Kaiser–Meyer–Olkin measure of 0.894.

**Results:**

This study included 2448 university students, with 1489 (60.8%) females. The median scores for KAP were 20 (Interquartile Range (IQR): 17–21), 26 (IQR: 23–28), and 35 (IQR: 32–38), respectively. Multivariate regression analysis indicated that being a junior (odds ratio [OR] = 0.720, 95% Confidence Interval (CI): 0.538–0.965, *p* = .028), senior or above (OR = 0.474, 95% CI: 0.325–0.691, *p* < .001), having divorced parents (OR = 0.618, 95% CI: 0.409–0.933, *p* = .022), having direct relatives with depression (OR = 0.710, 95% CI: 0.589–0.856, *p* < .001), and lacking intimate friends (OR = 0.344, 95% CI: 0.245–0.484, *p* < .001) were negatively associated with practice. Only having an attitude score of ≥26 (OR = 5.076, 95% CI: 4.230–6.091, *p* < .001) was significantly and positively associated with practice.

**Conclusion:**

University students had insufficient knowledge, positive attitude, and passive practice toward depression. Clinical interventions should focus on enhancing the understanding and management of depression among university students, particularly through targeted educational programs and support groups, to bridge the gap between knowledge and practice and foster a proactive approach to mental health care.

## Graphical abstract

This research summarizes a survey of 2448 students from 14 universities in Shandong Province. The survey assessed knowledge, attitude, and practice toward depression. Results show insufficient knowledge, positive attitudes, and passive practices. Factors negatively affecting practice include being a junior or senior, having divorced parents, having relatives with depression, and lacking close friends. A positive attitude was strongly associated with better practices. The study highlights the need for targeted education and support to improve depression management among students.

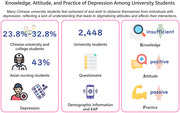

## 1 INTRODUCTION

Depression, a significant mental health issue, is seeing an increasing prevalence globally (Coenen et al., 2018; Matsuo et al., 2019). Globally, it is estimated that over 264 million people of all ages suffer from depression (Martínez‐Castaño et al., 2020), marking it as a profound psychological disorder. It impairs emotions, thoughts, and behaviors, leading to a decreased quality of life and substantial societal and economic burdens (Dolotov et al., 2022; Gao et al., 2018). The incidence of depression is particularly rising among the youth, especially university students (Ibrahim et al., 2013; Rotenstein et al., 2016). For instance, a study indicated that the overall prevalence of depression among Chinese university and college students was 23.8% (Lei et al., 2016). Furthermore, a cross‐sectional survey conducted to assess depressive symptoms among college students in Liaoning, China, found that the prevalence of depression among 1362 students was 32.8% (Dong & Li, 2020). Additionally, among Asian nursing students, the prevalence rates of depression and moderate to severe anxiety are 43% and 56%, respectively (Savitsky et al., 2020; Tung et al., 2018). This demographic contends with various stressors, including academic pressures, interpersonal challenges, and future uncertainties, increasing their susceptibility to depression (van der Wal et al., 2021; Zhang et al., 2022). Therefore, focused research on depression in university students is essential to understand their mental health better and to develop appropriate support and interventions.

A recent study has discovered that a significant proportion of university students in China exhibit feelings of shame toward individuals with depression and express a desire to maintain social distance from them (He et al., 2021). This lack of understanding about depression among university students can lead to stigmatizing attitudes, which may subsequently impact their practices and interactions with affected individuals. Knowledge, attitude, and practice (KAP) surveys are instrumental research tools for evaluating individuals' understanding, viewpoints, and behaviors on specific subjects (Aerts et al., 2020; Takeuchi et al., 2019). In depression studies, KAP surveys elucidate university students' awareness, attitudes, and practices concerning depression. They facilitate a comprehensive analysis of students' comprehension of depression, their perspectives on it, and their responses to depressive episodes. Despite their importance, there is a notable scarcity of such KAP studies in China.

The aim of this study is to delve into university students' KAP regarding depression via a KAP survey and to provide scientific evidence for the development of targeted mental health interventions. This research is crucial as it contributes to improving the mental health of college students and promotes psychosocial well‐being by shedding light on critical aspects of mental health awareness, stigma, and management among this vulnerable population. This investigation will guide the development and implementation of tailored interventions designed to enhance mental health outcomes. Furthermore, to validate the KAP theoretical framework, this study additionally explores the relationships between KAP regarding depression among university students.

## 2 MATERIALS AND METHODS

### 2.1 Study design and participants

This cross‐sectional study was conducted in a selection of universities randomly chosen across Shandong Province, spanning from October 25 to November 8, 2023. It specifically targeted the student population within these universities. The research protocol for this study received approval from the Ethics Committee of the Shandong Provincial Mental Health Center (Approval Number: 2022‐Yan‐Lun‐Shen‐85), thereby ensuring compliance with established ethical guidelines. Prior to participation, informed consent was obtained from all respondents.

Students enrolled in the 2019–2023 academic cohorts across colleges and universities, encompassing junior college students, undergraduates, and graduate students, were included in the study. Key exclusion criteria included (1) questionnaires with completion times below 86 s or exceeding 1800 s, to ensure adequate response consideration and data integrity, and (2) incomplete questionnaires or those with participant ages outside the 16–40 years range, or with missing or irregular entries.

Out of the 2535 questionnaires initially collected, 55 were excluded due to inappropriate completion times, and an additional 32 were excluded based on age criteria and incomplete or abnormal responses. Consequently, the final analysis comprised 2448 valid questionnaires, offering a comprehensive overview of the targeted demographic group.

### 2.2 Questionnaire introduction

The questionnaire was meticulously designed based on insights from previously published literature (Lee et al., 2020; Zakhour et al., 2020; Zuckerbrot et al., 2018) and underwent thorough revisions incorporating feedback from several experts in the pertinent field. It was then pilot‐tested with a select group of respondents (51 in number) to evaluate its reliability, yielding a Cronbach's alpha of 0.816, indicative of high reliability. After this initial assessment, the questionnaire was administered to a broader population. The total scale demonstrated a reliability of 0.760, while the subscales for knowledge, attitude, and behavior showed reliabilities of 0.827, 0.721, and 0.724, respectively.

The finalized questionnaire, presented in Chinese, is structured into four dimensions designed to comprehensively assess aspects related to depression. The first dimension focuses on demographic information, featuring 16 questions about participant characteristics. The knowledge dimension comprises nine questions addressing symptoms, causes, and related disorders of depression. Scoring in this dimension varies, with multiple‐choice questions allowing cumulative scoring for correct answers and 0 points for incorrect ones; one open question allows for a maximum of 1 point for correct but unclear or incorrect multiple‐choice responses, leading to a possible score range of 2–23 points. The Attitude Dimension includes seven questions, evaluating attitudes toward individuals with depression and treatment approaches. Except for one open question, the remaining items utilize a 5‐point Likert scale, assigning 1–5 points based on attitude intensity, and scores can range from 6 to 30 points. Lastly, the practice dimension contains 11 questions on personal experiences and coping mechanisms, with six and 11 being open questions. The other items also use a 5‐point Likert scale, where points from 1 to 5 are assigned according to the degree of action, resulting in a total score range of 9–45 points.

We employed the Average Score Method to calculate the difficulty coefficients for our study on depression‐related KAP. The specific coefficients of difficulty for each item are detailed in the Supporting Information.

### 2.3 Survey deployment details

Between September and November 2023, a comprehensive study was carried out in Shandong Province, targeting various universities including Shandong University, Shandong University of Finance and Economics, Shandong Normal University, and other institutions such as Shandong Jianzhu University, Qingdao University, and several more, totaling 14 universities. This study involved students from cohorts ranging from 2019 to 2023, encompassing junior college, undergraduate, and graduate students. To select the universities, a list of all institutions in Shandong Province meeting our research criteria was compiled. A random number generator was then employed to randomly select universities, ensuring each had an equal opportunity to be chosen, which is crucial for maintaining a representative and unbiased sample. Following the selection of universities, secondary colleges within these universities were also randomly chosen. Within each selected secondary college, a stratified random cluster sampling method was implemented. Specifically, 1–2 classes from each grade were randomly selected. The method of questionnaire distribution involved teachers handing out electronic questionnaires in classrooms or sending them to WeChat groups of the selected classes. Students voluntarily completed the questionnaires at their discretion. All data collected were rigorously anonymized to protect the personal identities and privacy of the participants.

### 2.4 Statistical analyses

Descriptive analyses were performed on demographic data and dimension scores, using means and standard deviations for normal distributions, and medians and interquartile ranges for non‐normal distributions. Categorical data were expressed as counts and percentages. The average score method was used to calculate the difficulty coefficient of each question. Score differences across demographics were analyzed using *t*‐tests or analysis of variance for normal, and Wilcoxon–Mann–Whitney or Kruskal–Wallis tests for non‐normal data. Correlations used Pearson or Spearman coefficients based on data normality. Univariate and multivariate logistic regression were performed to explore the risk factors associated with K, A, and P, Univariate variables with *p* < .1 and *p* < .25 were enrolled in multivariate regression. All *p*‐values were reported to three decimal places, with significance determined at *p* < .05.

## 3 RESULTS

### 3.1 Demographic characteristics

The normality test showed that the scores distribution of each dimension deviated from normality (all of *p* < .001) (as shown in Table S1). Of the 2448 university students who participated in this study, 1489 (60.8%) were female, 1141 (46.6%) were not >18 years old, 1627 (66.5%) were freshman, and 1763 (72.0%) were not the only children. In the meantime, 958 (39.1%) had parents who graduated from junior high school, 1966 (80.3%) were single, 1463 (59.8%) often participated in extracurricular activities, 807 (33.0%) participated in one or two times of group activities per semester, and 2228 (91.0%) had intimate friends. The median (25th percentile, 75th percentile) score of knowledge, attitude, and practice were 20 (17,21), 26 (23,28), and 35 (32,38), separately. Analyses of differences in demographic characteristics showed that participants with different gender, age, registered residence, grade, co‐residents, family annual income, parents' education, major, participation in extracurricular activities, and frequency of participation in group activities were more likely to have different knowledge scores. Concurrently, participants with different age, grade, co‐residents, parents' marital status, lived with parents before enrolling, parents' education, major, direct relatives with depression, participation in extracurricular activities, frequency of participation in group activities, and presence of intimate friends were more likely to have different attitude scores. Furthermore, participants with different age, grade, co‐residents, parents' marital status, lived with parents before enrolling, family annual income, major, direct relatives with depression, relationship status, participation in extracurricular activities, frequency of participation in group activities, and presence of intimate friends were more likely to have different practice scores (all of *p* < .005) (as shown in Table 1). With regard to the overall distribution of scores, 51.4%, 51.8%, and 56.4% had scores higher than or equal to the median for knowledge, attitude, and practice scores, respectively (as shown in Table 2).

**TABLE 1:** Baseline characteristics.

Variable	N (%)	Knowledge score		Attitude score		Practice score	
		Median (25th percentile, 75th percentile)	*p*	Median (25th percentile, 75th percentile)	*p*	Median (25th percentile, 75th percentile)	*p*
**Total**	2448	20 (17,21)		26 (23,28)		35 (32,38)	
**Gender**			**<.001**		.145		0.474
Male	959 (39.2)	19 (16,21)		26 (22,28)		35 (32,39)	
Female	1489 (60.8)	20 (17,21)		26 (23,28)		35 (32,38)	
**Age (years)**	19 (18,19)	–	**/**	/	/	/	/
**Age group (years)**			**<.001**		**.015**		**<.001**
≤18	1141 (46.6)	20 (17,22)		26 (23,29)		36 (32,39)	
18–20	998 (40.8)	19 (16,21)		26 (23,28)		35 (32,38)	
>20	309 (12.6)	19 (15,21)		25 (22,28)		34 (31,37)	
**Registered residence**			**<.001**		.424		.337
Rural	1653 (67.5)	19 (16,21)		26 (23,28)		35 (32,38)	
Non‐rural	795 (32.5)	20 (17,22)		26 (23,28)		35 (32,39)	
**Grade**			**<.001**		**<.001**		**<.001**
Freshman	1627 (66.5)	20 (17,22)		26 (23,29)		36 (32,39)	
Sophomore	397 (16.2)	19 (15,21)		25 (23,28)		35 (32,38)	
Junior	270 (11.0)	19 (15,21)		25 (22,28)		34 (31,38)	
Senior and above	154 (6.3)	20 (15,22)		24.5 (22,27)		33 (30,36)	
**Co‐residents**			**.036**		**.004**		**.006**
1 person	121 (4.9)	19 (15,21)		24 (22,27)		33 (31,37)	
2 people	740 (30.2)	20 (17,21)		26 (23,28)		35 (32,39)	
3 people	989 (40.4)	20 (17,21)		26 (23,28)		35 (32,39)	
4 people	421 (17.2)	19 (16,21)		26 (23,28)		35 (32,38)	
5 people and above	177 (7.2)	20 (16,21)		26 (24,29)		35 (32,38)	
**Parents’ marital status**			.669		**.003**		**<.001**
Married	2231 (91.1)	20 (17,21)		26 (23,28)		35 (32,39)	
Divorced	125 (5.1)	20 (16,21)		24 (22,27)		32 (29,37)	
Other	92 (3.8)	19 (15.5,21)		25 (22.5,28)		34 (31,37.5)	
**Only child**			.063		.064		.411
Yes	685 (28.0)	20 (17,22)		25 (23,28)		35 (32,39)	
No	1763 (72.0)	20 (17,21)		26 (23,28)		35 (32,38)	
**Lived with parents before enrolling**			.168		**<.001**		**<.001**
Yes	2317 (94.6)	20 (17,21)		26 (23,28)		35 (32,39)	
No	131 (5.4)	19 (16,21)		24 (22,27)		34 (30,37)	
**Family annual income ，Chinese Yuan (CNY**)			**.002**		.051		**.025**
<10,000	201 (8.2)	19 (14,21)		26 (22,27)		35 (31,38)	
10,000–50,000	834 (34.1)	19 (17,21)		25 (23,28)		35 (32,38)	
50,000–100,000	674 (27.5)	20 (17,22)		26 (23,28)		35 (32,38)	
100,000–200,000	539 (22.0)	20 (17,22)		26 (23,29)		35 (32,39)	
>200,000	200 (8.2)	20 (15.5,22)		26 (23,28.5)		36 (33,39)	
**Parents’ education**			**<.001**		**.045**		.208
Elementary school and below	207 (8.5)	19 (16,21)		24 (22,28)		35 (32,38)	
Junior high school	958 (39.1)	19 (16,21)		26 (23,28)		35 (32,38)	
High school	589 (24.1)	19 (16,21)		26 (23,28)		36 (32,39)	
College	331 (13.5)	20 (17,22)		26 (23,28)		35 (32,39)	
Bachelor's degree and above	363 (14.8)	20 (18,22)		26 (23,28)		35 (32,39)	
**Major**			**<.001**		**.022**		**.010**
Engineering	513 (21.0)	20 (16,21)		26 (23,29)		36 (32,39)	
Medical	433 (17.7)	20 (18,22)		25 (22,28)		35 (32,38)	
Economics/management	1231 (50.3)	19 (16,21)		26 (23,28)		35 (32,38)	
Other	271 (11.1)	19 (15,21)		26 (23,28)		36 (32,39)	
**Direct relatives with depression**			.410		**.003**		**<.001**
Yes	66 (2.7)	20 (18,21)		24 (22,26)		30 (27,35)	
No	2382 (97.3)	20 (17,21)		26 (23,28)		35 (32,39)	
**Relationship status**			.099		.336		**.034**
Single	1966 (80.3)	20 (17,21)		26 (23,28)		35 (32,39)	
In a romantic relationship	464 (19.0)	20 (16,21)		26 (23,28)		35 (32,38)	
Other	18 (0.7)	17.5 (10,21)		25 (22,26)		33 (29,37)	
**Participation in extracurricular activities**			**.018**		**<.001**		**<.001**
Yes	1463 (59.8)	20 (17,22)		26 (24,29)		36 (33,39)	
No	985 (40.2)	19 (16,21)		24 (22,27)		34 (31,37)	
**Frequency of participation in group activities**			**<.001**		**<.001**		**<.001**
1–2 times per semester	807 (33.0)	19 (15,21)		25 (22,28)		35 (32,38)	
3–5 times per semester	745 (30.4)	20 (16,21)		26 (24,28)		35 (32,39)	
6–10 times per semester	338 (13.8)	21 (18,22)		26 (24,29)		36 (33,40)	
10 ‐times and more per semester	323 (13.2)	20 (18,22)		27 (24,29)		37 (33,39)	
Never	235 (9.6)	19 (17,21)		24 (20,26)		34 (30,37)	
**Presence of intimate friends**			.166		**<.001**		**<.001**
Yes	2228 (91.0)	20 (17,21)		26 (23,28)		36 (32,39)	
No	220 (9.0)	19 (17,21)		22 (20,25)		32 (29,35)	

Significance of bold value p<0.05.

**TABLE 2:** Score distribution.

	Mean	Median	25th percentile	75th percentile	Minimum	Maximum	<median N (%)	≥median N (%)
Knowledge score	18.3	20	17	21	2	23	1189 (48.6)	1259 (51.4)
Attitude score	25.2	26	23	28	6	30	1179 (48.2)	1269 (51.8)
Practice score	35.1	35	32	38	9	45	1068 (43.6)	1380 (56.4)

### 3.2 Knowledge, attitude, and practice

First, we tested the difficulty coefficient of each dimension of the problem, and the results showed that the difficulty coefficient of the knowledge dimension was 0.41–0.88, the difficulty coefficient of the attitude dimension was 0.68–0.90, and the difficulty coefficient of the practice dimension was 0.63–0.95 (as shown in Table S1). The knowledge dimension scores showed that 83.4% believed that “Depression, in turn, can lead to greater stress and functional impairment, affecting the patient's life and exacerbating depressive symptoms” (K7) is correct; on the other hand, 40.9% believe that “women are more likely to suffer from depression than men.” (K4) is incorrect. Further, 94.2% believe that the main symptom of depression is low mood (K1), 93.8% believe that university students are susceptible to depression because of excessive academic stress (K3), 88.7% believe that the general university students should differentiate between depression and general moodiness by the extent of its impact on their life (K5), and 78.3% believe that tumors may cause depression (K6) (as shown in Table 3).

**TABLE 3:** Knowledge dimension score.

	A correct	B incorrect	C uncertain
**1. Depression is closely related to genetic factors, often seen in first‐degree relatives of depressed patients, with a high concordance rate in monozygotic twins**.	**1174 (48.0)**	305 (12.5)	969 (39.6)
2**. Women are more likely to suffer from depression than men**.	**1001 (40.9)**	503 (20.5)	944 (38.6)
3**. Depression, in turn, can lead to greater stress and functional impairment, affecting the patient's life and exacerbating depressive symptoms**.	**2042 (83.4)**	52 (2.1)	354 (14.5)
**4. Patients with cardiovascular diseases, cancer, diabetes, and respiratory system diseases are more prone to depression**.	**1540 (62.9)**	232 (9.5)	676 (27.6)
**5. Depression can be treated with psychotherapy and medication**.	**2148 (87.7)**	46 (1.9)	254 (10.4)
**6. What** **are the main symptoms commonly associated with depression? (Multiple choices allowed)**	**N (%)**		
a. Low mood	**2305 (94.2)**		
b. Loss of interest and pleasure	2181 (89.1)		
c. Decreased energy leading to increased fatigue and reduced activity	2058 (84.1)		
d. Decreased attention	2042 (83.4)		
e. Reduced self‐esteem and confidence	2119 (86.6)		
f. Guilt feelings and a sense of worthlessness	2069 (84.5)		
g. Pessimistic thoughts about the future	2073 (84.7)		
h. Sleep disturbances	2060 (84.2)		
i. Decreased appetite	1950 (79.7)		
**7. What are the reasons that make college students prone to depression? (Multiple choices allowed)s**	**N (%)**		
a. Excessive academic stress	**2297 (93.8)**		
b. Interpersonal relationship issues	2258 (92.2)		
c. Employment pressure	2134 (87.2)		
d. Setting excessively high personal expectations	1953 (79.8)		
e. Family relationships	2032 (83.0)		
f. Emotional problems	2014 (82.3)		
**8. How should ordinary college students differentiate between depression and general mood fluctuations? (Multiple choices allowed)**	**N (%)**		
a. Duration of emotional state	2157 (88.1)		
b. Intensity and frequency of symptoms	2149 (87.8)		
c. Degree of impact on daily life	**2171 (88.7)**		
d. Other	26 (1.1)		
**9. Which of the following diseases may contribute to depression? (Multiple choices allowed)**	**N (%)**		
a. Influenza virus	1011 (41.3)		
b. Tumors	**1918 (78.3)**		
c. Systemic lupus erythematosus	1750 (71.5)		
d. Heart disease	1728 (70.6)		

Bold has the largest number of people.

When it comes to related attitudes, 62.1% strongly agree that mental health education plays an important role on university campuses (A3), and 56.8% say they are very willing to take the initiative to help friends or classmates when they have symptoms of depression (A1). In addition, regarding sharing their emotional and psychological problems with others, 32.6% were very willing, while 31.8% were neutral. It is worth noting that 31.9% were unsure if they might be suffering from depression (A5). On a relatively positive note, 47.9% strongly disagreed that depression is something to be ashamed of or to be stigmatized (A7) (as shown in Table 4).

**TABLE 4:** Attitude dimension score.

	Very willing	Willing	Neutral	Unwilling	Very unwilling
**1. If you observe symptoms of depression in friends or classmates, would you proactively help them?**	**1390 (56.8)**	650 (26.6)	337 (13.8)	49 (2.0)	22 (0.9)
**2. Are you willing to share your own emotions and psychological issues with others?**	**799 (32.6)**	638 (26.1)	779 (31.8)	174 (7.1)	58 (2.4)
	**Very important**	Important	Average	Unimportant	Very unimportant
**3. Do you consider the role of mental health education on university campuses important?**	**1519 (62.1)**	655 (26.8)	236 (9.6)	20 (0.8)	18 (0.7)
	**Completely agree**	Agree	Neutral	Disagree	Completely disagree
**4. Do you believe you can get along well with others in your family, society, and school?**	**1098 (44.9)**	864 (35.3)	419 (17.1)	46 (1.9)	21 (0.9)
**5. Do you think you might suffer from depression?**	160 (6.5)	370 (15.1)	**780 (31.9)**	632 (25.8)	506 (20.7)
**6. Regarding the occurrence of depression, do you believe you can acknowledge and face it, actively seeking help or finding ways to overcome it?**	**1125 (46.0)**	831 (33.9)	426 (17.4)	46 (1.9)	20 (0.8)
**7. Depression is something shameful or stigmatized**.	114 (4.7)	93 (3.8)	294 (12.0)	774 (31.6)	**1173 (47.9)**

Bold has the largest number of people.

Participants' responses to the practice dimension items indicated that 82.2% had never engaged in non‐suicidal self‐injurious behavior (P10), and 78.9% had never been dependent on or cathartic with alcohol (P7). However, 33.6% rarely vented their negative emotions through self‐relief methods (P4). In addition, 44.2% reported that they sometimes felt stress from family, school, and society (P3), and 37.7% indicated that their university sometimes conducted depression‐related publicity or educational activities (P1) (as shown in Table 5).

**TABLE 5:** Practice dimension score.

	Almost never	Rarely	Sometimes	Often	Always
**1. Is there frequent promotion or educational activities related to depression at your school?**	242 (9.9)	319 (13.0)	**924 (37.7)**	677 (27.7)	286 (11.7)
**2. Do you often experience symptoms of depression?**	**1102 (45.0)**	717 (29.3)	533 (21.8)	68 (2.8)	28 (1.1)
**3. Do you often feel stress from family, school, society, etc.?**	460 (18.8)	534 (21.8)	**1083 (44.2)**	287 (11.7)	84 (3.4)
**4. Do you often use self‐relief methods to vent negative emotions?**	619 (25.3)	**822 (33.6)**	784 (32.0)	179 (7.3)	44 (1.8)
**5**. I**n situations** **where self‐relief of negative emotions is not ideal, do you actively seek help?**	252 (10.3)	380 (15.5)	**877 (35.8)**	639 (26.1)	300 (12.3)
**6. Do you have alcohol dependence and often use alcohol to release emotions?**	**1931 (78.9)**	301 (12.3)	166 (6.8)	37 (1.5)	13 (0.5)
**7. Do you often feel physically uncomfortable?**	**1012 (41.3)**	778 (31.8)	530 (21.7)	102 (4.2)	26 (1.1)
**8. Do you feel anxious or nervous because of physical discomfort?**	**931 (38.0)**	671 (27.4)	672 (27.5)	141 (5.8)	33 (1.3)
**9. Have you ever engaged in non‐suicidal self‐harm behaviors?**	**2012 (82.2)**	280 (11.4)	122 (5.0)	22 (0.9)	12 (0.5)
**10. If you encounter psychological issues, which methods are you more inclined to choose for seeking help? (Multiple choices allowed)**	**N (%)**				
a. Psychological counseling	1396 (57.0)				
b. Self‐regulation	**2121 (86.6)**				
c. Confiding in or seeking help from classmates	1652 (67.5)				
d. Confiding in or seeking help from teachers or counselors	947 (38.7)				
e. Confiding in friends or family	1542 (63.0)				
f. Participating in mental health activities	929 (37.9)				
g. Other	41 (1.7)				
**11. From which channels do you generally acquire knowledge about mental health? (Multiple choices allowed)**	**N (%)**				
a. School promotions and campus lectures	**2015 (82.3)**				
b. Internet searches	1965 (80.3)				
c. Library books and journal articles	1582 (64.6)				
d. Mental health apps	1530 (62.5)				
e. Courses related to psychology or mental health education	1630 (66.6)				
f. Recommendations from teachers, classmates, and friends	1378 (56.3)				
g. Student club activities	1194 (48.8)				
h. Other	14 (0.6)				

Bold has the largest number of people.

### 3.3 Correlation analysis and multivariate regression analysis

In the correlation analysis, significant positive correlations were found between knowledge and attitude (*r* = 0.154, *p* < .001), knowledge and practice (*r* = 0.060, *p* = .003), and attitude and practice (*r* = 0.557, *p* < .001), respectively (as shown in Table 6).

**TABLE 6:** Correlation analysis.

	Knowledge	Attitude	Practice
**Knowledge**	1.000	0.154 (*p*<.001)	0.060 (*p* = .003)
**Attitude**	0.154 (*p*<.001)	1.000	0.557 (*p*<.001)
**Practice**	0.060 (*p* = .003)	0.557 (*p*<.001)	1.000

Multivariate regression analysis indicated significant associations with knowledge levels (as shown in Table 7, 8, 9)Females (odds ratio [OR] = 1.363, 95% CI: [1.130, 1.644], *p* = .001) (reference: Male) and individuals with non‐rural registered residence (OR = 1.304, 95% CI: [1.093, 1.555], *p* = .003) (reference: Rural) were more likely to have higher knowledge scores. Additionally, majors in medical fields (OR = 1.416, 95% CI: [1.076, 1.862], *p* = .013) and economics/management (OR = 0.763, reference: engineering, 95% CI: [0.603, 0.966], *p* = .025) (reference: Engineering) showed distinct patterns of knowledge. Frequent participation in group activities also related to better knowledge, with 6–10 participations per semester (OR = 1.992, 95% CI: [1.413, 2.809], *p* < .001) and >10 times per semester (OR = 1.442, 95% CI: [1.024, 2.030], *p* = .036) significantly associated with higher scores (reference: Never).

**TABLE 7:** Univariate and multivariate analysis for knowledge dimension.

Cutoff value:≥20/<20	No.	Univariate		Multivariate (forward, *p*<.1)	
		OR (95%CI)	*P*	OR (95%CI)	*p*
**Gender**					
Male	467/959	Ref.		Ref.	
Female	792/1489	1.197 (1.018,1.408)	**.030**	1.363 (1.130,1.644)	**.001**
**Age group (years)**					
≤18	639/1141	Ref.			
18–20	474/998	0.711 (0.599,0.843)	**<.001**		
>20	146/309	0.704 (0.547,0.905)	**.006**		
**Registered residence**					
Rural	800/1653	Ref.		Ref.	
Non‐rural	459/795	1.457 (1.228,1.728)	**<.001**	1.304 (1.093,1.555)	**.003**
**Grade**					
Freshman	884/1627	Ref.			
Sophomore	178/397	0.683 (0.548,0.852)	**.001**		
Junior	116/270	0.633 (0.488,0.821)	**.001**		
Senior and above	81/154	0.933 (0.670,1.299)	.680		
**Co‐residents**					
1 person	57/121	Ref.			
2 people	403/740	1.343 (0.913,1.974)	.134		
3 people	513/989	1.210 (0.829,1.766)	.323		
4 people	197/421	0.987 (0.659,1.481)	.951		
5 people and above	89/177	1.136 (0.715,1.804)	.590		
**Parents’ marital status**					
Married	1151/2231	Ref.			
Divorced	65/125	1.017 (0.709,1.458)	.929		
Other	43/92	0.823 (0.542,1.251)	.362		
**Only child**					
Yes	377/685	Ref.			
No	882/1763	0.818 (0.685,0.976)	**.026**		
**Lived with parents before enrolling**					
Yes	1202/2317	Ref.			
No	57/131	0.715 (0.501,1.019)	.063		
**Family annual income (CNY)**					
<10,000	90/201	Ref.			
10,000–50,000	407/834	1.176 (0.863,1.602)	.306		
50,000–100,000	354/674	1.364 (0.994,1.872)	.054		
100,000–200,000	305/539	1.608 (1.160,2.227)	**.004**		
>200,000	103/200	1.310 (0.884,1.939)	.178		
**Parents’ education**					
Elementary school and below	89/207	Ref.			
Junior high school	478/958	1.320 (0.975,1.787)	.072		
High school	284/589	1.235 (0.897,1.699)	.196		
College	190/331	1.787 (1.258,2.538)	**.001**		
Bachelor's degree and above	218/363	1.993 (1.410,2.818)	**<.001**		
**Major**					
Engineering	263/513	Ref.		Ref.	
Medical	275/433	1.654 (1.274,2.148)	**<.001**	1.416 (1.076,1.862)	**.013**
Economics/management	588/1231	0.869 (0.707,1.068)	.183	0.763 (0.603,0.966)	**.025**
Other	133/271	0.916 (0.682,1.230)	.560	0.797 (0.582,1.092)	.158
**Direct relatives with depression**					
Yes	41/66	1.567 (0.947,2.594)	.080		
No	1218/2382	Ref.			
**Relationship status**					
Single	1017/1966	Ref.			
In a romantic relationship	236/464	0.966 (0.789,1.183)	.737		
Other	6/18	0.467 (0.174,1.248)	.129		
**Participation in extracurricular activities**					
Yes	777/1463	Ref.			
No	482/985	0.846 (0.720,0.995)	**.043**		
**Frequency of participation in group activities**					
1–2 times per semester	358/807	0.906 (0.677,1.213)	.507	0.931 (0.693,1.250)	.633
3–5 times per semester	388/745	1.235 (0.921,1.657)	.159	1.240 (0.921,1.669)	.157
6–10 times per semester	218/338	2.064 (1.470,2.899)	**<.001**	1.992 (1.413,2.809)	**<.001**
10 times and more per semester	185/323	1.523 (1.086,2.136)	**.015**	1.442 (1.024,2.030)	**.036**
Never	110/235	Ref.		Ref.	
**Presence of intimate friends**					
Yes	1155/2228	Ref.			
No	104/220	0.833 (0.631,1.099)	.196		

Abbreviation: OR, odds ratio.

Significance of bold value p<0.05.

**TABLE 8:** Univariate and multivariate analysis for attitude dimension.

Cutoff value:≥26/<26	No.	Univariate		Multivariate (forward, *p*<.1)	
		OR (95%CI)	*p*	OR (95%CI)	*p*
**Gender**					
Male	492/959	Ref.			
Female	777/1489	1.036 (0.881,1.218)	.671		
**Age group (years)**					
≤18	606/1141	Ref.			
18–20	520/998	0.960 (0.810,1.139)	.642		
>20	143/309	0.761 (0.591,0.979)	**.033**		
**Registered residence**					
Rural	866/1653	Ref.			
Non‐rural	403/795	0.934 (0.789,1.107)	.431		
**Grade (adjusted)**					
Freshman	882/1627	Ref.			
Sophomore	195/397	0.815 (0.655,1.016)	.069		
Junior	129/270	0.773 (0.597,1.000)	.050		
Senior and above	63/154	0.585 (0.418,0.818)	**.002**		
**Co‐residents**					
1 person	50/121	Ref.			
2 people	376/740	1.467 (0.994,2.165)	.054		
3 people	520/989	1.574 (1.074,2.309)	**.020**		
4 people	222/421	1.584 (1.052,2.385)	**.028**		
5 people and above	101/177	1.887 (1.181,3.015)	**.008**		
**Parents’ marital status**					
Married	1177/2231	Ref.			
Divorced	52/125	0.638 (0.443,0.919)	**.016**		
Other	40/92	0.689 (0.452,1.049)	.082		
**Only child**					
Yes	341/685	Ref.			
No	928/1763	1.121 (0.940,1.338)	.204		
**Lived with parents before enrolling**					
Yes	1216/2317	Ref.			
No	53/131	0.615 (0.430,0.880)	**.008**		
**Family annual income (CNY)**					
<10,000	101/201	Ref.			
10,000–50,000	408/834	0.948 (0.697,1.290)	.735		
50,000–100,000	360/674	1.135 (0.828,1.556)	.431		
100,000–200,000	287/539	1.128 (0.815,1.559)	.468		
>200,000	113/200	1.286 (0.868,1.905)	.210		
**Parents’ education**					
Elementary school and below	89/207	Ref.			
Junior high school	496/958	1.423 (1.051,1.927)	**.022**		
High school	316/589	1.535 (1.115,2.112)	**.009**		
College	179/331	1.561 (1.100,2.216)	**.013**		
Bachelor's degree and above	189/363	1.440 (1.021,2.031)	**.038**		
**Major**					
Engineering	290/513	Ref.		Ref.	
Medical	197/433	0.642 (0.496,0.830)	**.001**	0.630 (0.479,0.828)	**.001**
Economics/management	631/1231	0.809 (0.657,0.995)	**.045**	0.874 (0.701,1.089)	.229
Other	151/271	0.968 (0.719,1.302)	.828	0.999 (0.731,1.366)	.995
**Direct relatives with depression**					
Yes	24/66	0.522 (0.314,0.867)	**.012**	0.503 (0.296,0.854)	**.011**
No	1245/2382	Ref.		Ref.	
**Relationship status**					
Single	1012/1966	Ref.			
In a romantic relationship	249/464	1.092 (0.891,1.337)	.396		
Other	8/18	0.754 (0.296,1.919)	.554		
**Participation in extracurricular activities**					
Yes	890/1463	Ref.		Ref.	
No	379/985	0.403 (0.341,0.475)	**<.001**	0.521 (0.428,0.635)	**<.001**
**Frequency of participation in group activities**					
1–2 times per semester	368/807	1.788 (1.315,2.432)	**<.001**	1.257 (0.907,1.742)	.170
3–5 times per semester	410/745	2.611 (1.915,3.560)	**<.001**	1.496 (1.060,2.111)	**.022**
6–10 times per semester	208/338	3.413 (2.403,4.849)	**<.001**	1.695 (1.141,2.516)	**.009**
10 times and more per semester	208/323	3.859 (2.701,5.511)	**<.001**	2.010 (1.339,3.018)	**.001**
Never	75/235	Ref.		Ref.	
**Presence of intimate friends**					
Yes	1221/2228	Ref.		Ref.	
No	48/220	0.230 (0.165,0.320)	**<.001**	0.253 (0.179,0.356)	**<.001**
**Knowledge score**					
<20	551/1189	Ref.		Ref.	
≥20	718/1259	1.537 (1.310,1.802)	**<.001**	1.523 (1.286,1.805)	**<.001**

Abbreviation: OR, odds ratio.

Significance of bold value p<0.05.

**TABLE 9:** Univariate and multivariate analysis for practice dimension.

Cutoff value:≥35/<35	No.	Univariate		Multivariate (forward, *p*<.1)	
		OR (95%CI)	*p*	OR (95%CI)	*p*
**Gender**					
Male	540/959	Ref.			
Female	840/1489	1.004 (0.853,1.183)	.959		
**Age group (years)**					
≤18	685/1141	Ref.			
18–20	553/998	0.827 (0.696,0.983)	**.031**		
>20	142/309	0.566 (0.439,0.729)	**<.001**		
**Registered residence**					
Rural	950/1653	Ref.			
Non‐rural	430/795	0.872 (0.735,1.034)	.114		
**Grade**					
Freshman	977/1627	Ref.		Ref.	
Sophomore	210/397	0.747 (0.599,0.932)	**.010**	0.809 (0.631,1.037)	.094
Junior	134/270	0.656 (0.506,0.849)	**.001**	0.720 (0.538,0.965)	**.028**
Senior and above	59/154	0.413 (0.294,0.580)	**<.001**	0.474 (0.325,0.691)	**<.001**
**Co‐residents**					
1 person	53/121	Ref.			
2 people	417/740	1.656 (1.124,2.440)	**.011**		
3 people	576/989	1.789 (1.223,2.618)	**.003**		
4 people	238/421	1.669 (1.110,2.508)	**.014**		
5 people and above	96/177	1.521 (0.955,2.421)	.077		
**Parents’ marital status**					
Married	1286/2231	Ref.		Ref.	
Divorced	52/125	0.523 (0.363,0.754)	**.001**	0.618 (0.409,0.933)	**.022**
Other	42/92	0.617 (0.406,0.938)	**.024**	0.683 (0.427,1.092)	.112
**Only child**					
Yes	391/685	Ref.			
No	989/1763	0.961 (0.804,1.148)	.660		
**Lived with parents before enrolling**					
Yes	1325/2317	Ref.			
No	55/131	0.542 (0.379,0.774)	**.001**		
**Family annual income (CNY)**					
<10,000	101/201	Ref.			
10,000–50,000	454/834	1.183 (0.869,1.610)	.286		
50,000–100,000	390/674	1.360 (0.991,1.865)	.057		
100,000–200,000	310/539	1.340 (0.968,1.855)	.077		
>200,000	125/200	1.650 (1.108,2.457)	**.014**		
**Parents’ education**					
Elementary school and below	106/207	Ref.			
Junior high school	538/958	1.221 (0.903,1.649)	.194		
High school	341/589	1.310 (0.953,1.800)	.096		
College	186/331	1.222 (0.863,1.732)	.259		
Bachelor's degree and above	209/363	1.293 (0.918,1.822)	.142		
**Major**					
Engineering	309/513	Ref.			
Medical	230/433	0.748 (0.578,0.969)	**.028**		
Economics/management	688/1231	0.836 (0.678,1.032)	.095		
Other	153/271	0.856 (0.635,1.153)	.307		
**Direct relatives with depression**					
Yes	24/66	0.327 (0.192,0.556)	**<.001**	0.710 (0.589,0.856)	**<.001**
No	1245/2382	Ref.		Ref.	
**Relationship status**					
Single	1012/1966	Ref.			
In a romantic relationship	249/464	0.886 (0.723,1.086)	.245		
Other	8/18	0.479 (0.185,1.240)	.129		
**Participation in extracurricular activities**					
Yes	890/1463	Ref.			
No	379/985	0.479 (0.406,0.564)	**<.001**		
**Frequency of participation in group activities**					
1–2 times per semester	368/807	1.755 (1.305,2.360)	**<.001**		
3–5 times per semester	410/745	2.205 (1.634,2.976)	**<.001**		
6–10 times per semester	208/338	2.615 (1.857,3.683)	**<.001**		
10 times and more per semester	208/323	2.928 (2.068,4.147)	**<.001**		
Never	75/235	Ref.			
**Presence of intimate friends**					
Yes	1221/2228	Ref.		Ref.	
No	48/220	0.233 (0.170,0.319)	**<.001**	0.344 (0.245,0.484)	**<.001**
**Knowledge score**					
<20	651/1189	Ref.			
≥20	729/1259	1.137 (0.969,1.334)	.116		
**Attitude score**					
<26	412/1179	Ref.		Ref.	
≥26	968/1269	5.987 (5.020,7.141)	**<.001**	5.076 (4.230,6.091)	**<.001**

Abbreviation: OR, odds ratio.

Significance of bold value p<0.05.

In terms of attitudes (as detailed in Table 7), a major in medical fields was associated with a more negative attitude (OR = 0.630, 95% CI: [0.479, 0.828], *p* = .001) (reference: Engineering), as was having direct relatives with depression (OR = 0.503, 95% CI: [0.296, 0.854], *p* = .011) (reference: No). Lack of participation in extracurricular activities (OR = 0.521, 95% CI: [0.428, 0.635], *p* < .001) (reference: Yes) and absence of intimate friends (OR = 0.253, 95% CI: [0.179, 0.356], *p* < .001) (reference: Yes) were also linked to more negative attitudes. Conversely, participation in >2 group activities (OR > 1, *p* < .05) (reference: Never) and a knowledge score greater than or equal to 20 (OR = 1.523, 95% CI: [1.286, 1.805], *p* < .001) (reference: <20) were linked to more positive attitudes.

Regarding practices (refer to Table 7), students in their junior (OR = 0.720, 95% CI: [0.538, 0.965], *p* = .028) and senior years or above (OR = 0.474, 95% CI: [0.325, 0.691], *p* < .001) showed lower rates of positive practices (reference: Freshman). The presence of divorced parents (OR = 0.618, 95% CI: [0.409, 0.933], *p* = .022) (reference: Married) and having direct relatives with depression (OR = 0.710, 95% CI: [0.589, 0.856], *p* < .001) (reference: No) also correlated with negative practices. Lack of intimate friends was strongly associated with negative practices (OR = 0.344, 95% CI: [0.245, 0.484], *p* < .001) (reference: Yes). In contrast, having an attitude score greater than or equal to 26 was significantly associated with positive practice (OR = 5.076, 95% CI: [4.230, 6.091], *p* < .001) (reference: <26).

## 4 DISCUSSION

The study reveals that university students possess moderate knowledge and attitudes but exhibit passive practices regarding depression, highlighting the disparity between awareness and action in mental health.

The main findings of this study indicate that university students possess insufficient knowledge but maintain a positive attitude and demonstrate passive practice toward depression. These findings align with existing literature that emphasizes a general lack of mental health knowledge among university students (Cheng et al., 2021; Siddique et al., 2022). The positive attitude but passive practice could suggest that while students are open to the idea of mental health management, they may lack the tools or motivation to actively engage in practices that could mitigate depression.

In the inter‐group comparisons, gender differences were evident, with females scoring significantly higher in knowledge about depression than males. This aligns with prior research highlighting gender disparities in health literacy (Rababah et al., 2019). Among age groups, younger students (≤18 years), compared to those aged over 18 years, demonstrated higher knowledge and practice scores. This could be attributed to recent educational reforms that integrate mental health awareness into early education (Herbert, 2022). Students from non‐rural areas also exhibited higher knowledge scores than their rural counterparts. This observation underscores the persistent urban‐rural divide in access to mental health resources. To address this, it is recommended that mental health education for rural students be enhanced through tailored digital programs. Additionally, targeted interventions such as campaigns and support groups could address the reluctance often seen in male students toward seeking mental health assistance (Khan et al., 2020). Additionally, for male students who traditionally exhibit reluctance in seeking mental health assistance, universities should initiate campaigns and support groups. Freshmen exhibited higher scores across all categories, which might be due to recent exposure to university orientation programs that often include mental health education.

In addition to the demographic and educational factors examined, it is crucial to consider the context of the COVID‐19 pandemic, which may have significantly influenced the mental health landscape among university students. The pandemic has been associated with increased levels of stress, anxiety, and depression due to factors such as social isolation, uncertainty about the future, and changes in educational delivery methods (Yu et al., 2021; Zhu et al., 2023). These conditions could have heightened awareness of mental health issues, potentially affecting the KAP related to depression observed in our study.

The correlation analysis in this study indicated positive relationships between knowledge, attitude, and practice. This finding is in line with the cognitive‐affective‐behavioral model of psychological functioning, which posits that increased knowledge can lead to more favorable attitudes and, subsequently, more active practices (Giotakos, 2022; Mertens et al., 2021).

The multivariate regression analysis from the study highlights critical trends in university students' KAP toward depression, influenced by various demographic and social factors. Females, with an OR of 1.363, and students from non‐rural backgrounds, with an OR of 1.304, demonstrated higher knowledge levels regarding depression compared to their male and rural counterparts. This enhanced awareness likely stems from broader societal and educational exposures, which typically offer greater access to mental health resources. Interestingly, students majoring in medical fields displayed substantial knowledge (OR = 1.416 compared to non‐medical fields) but more negative attitudes toward depression (OR = 0.630 compared to non‐medical fields), potentially due to the clinical focus of their education, which could engender a more critical perspective on mental health issues, indicating a need for broader mental health education across all disciplines. Conversely, students from non‐health‐related majors, such as economics and management, evidenced significant knowledge deficits, with economics/management students showing lower knowledge (OR = 0.763) compared to their engineering peers, who served as the reference category. This observation underscores the imperative for expansive mental health education across all academic disciplines, ensuring that every student (Gao, 2022; Martinengo et al., 2022).

Moreover, active participation in group activities significantly bolstered both knowledge and attitudes, highlighting the critical role of social engagement in enhancing mental health literacy. Frequent engagement in such activities not only fosters awareness but also builds supportive networks that are vital for mental well‐being, with participation 6–10 times per semester correlating with higher knowledge scores (OR = 1.992). Our findings echo a previous study, which also reported the positive impact of social activities on mental health among university students, emphasizing the universal importance of such engagements (Lv et al., 2024).

Given these insights, it is clear that universities must adopt a holistic approach to mental health education. This approach should include integrating mental health topics into various curricula, extending targeted educational efforts to rural students who might lack initial exposure, and promoting an environment that encourages active participation in group activities related to mental health. Such initiatives would likely bridge the gaps in knowledge and attitudes, thereby fostering a more proactive stance toward mental health practices among students (Amarasuriya et al., 2018; Yokomitsu et al., 2020).

The knowledge dimension scores in this study highlight a mixed understanding of depression among university students. A notable finding is the high level of awareness that depression can be treated with psychotherapy and medication. However, there is a notable gap in understanding the genetic aspects of depression, as only 48.0% of students correctly identified its genetic links. The high recognition of traditional symptoms such as low mood and loss of interest indicates a basic awareness of depression's manifestations. Given these findings, universities should focus on enhancing knowledge about the less understood aspects of depression, particularly its genetic factors (Ilbay et al., 2022; Saji Parel et al., 2022). Educational programs can be designed to provide comprehensive information about mental health, including genetic predispositions, to bridge this knowledge gap. Additionally, incorporating interactive sessions and discussions in these programs could make the learning process more engaging and effective (Shen, 2020).

In the attitude dimension, the majority of students exhibited a willingness to help peers showing symptoms of depression, reflecting a positive and proactive stance toward mental health. However, only 32.6% were very willing to share their own emotions, indicating a potential stigma or discomfort in disclosing personal mental health issues. This reluctance could be linked to the stigma surrounding mental health, as highlighted in previous study, where students perceived mental health issues as a personal weakness (Auerbach et al., 2018; Croarkin & MacMaster, 2019). To improve this situation, universities should focus on creating a supportive environment where students feel safe to express their mental health concerns (Amarasuriya et al., 2018; de Anta et al., 2022). Campaigns and workshops that destigmatize mental health issues and encourage open conversations can be instrumental in changing these attitudes. Peer support programs can also be implemented, where students are trained to provide initial support and guidance to their fellow students (Lem et al., 2022; Qiu et al., 2022).

The practice dimension reveals that while there is some level of engagement in mental health‐related practices, there is room for improvement. For instance, the data show that a substantial number of students rarely or almost never participate in depression‐related educational activities. Furthermore, a significant proportion of students reported frequently experiencing symptoms of depression and stress, indicating a need for more practical coping strategies and support. Universities should therefore enhance the availability and visibility of mental health resources and activities on campus. Developing regular, engaging, and interactive mental health programs can encourage greater student participation (Mohammed et al., 2022; Yuan et al., 2021). Additionally, integrating mental health education into the core curriculum could ensure that all students receive essential information and skills to manage their mental health effectively. Providing accessible mental health services, such as counseling and support groups, can also offer practical support for students experiencing mental health issues.

Limitations of this study include its cross‐sectional design, which precludes the establishment of causality between the observed factors and depression KAP among university students. The reliance on self‐reported data may also introduce response biases, as students could overestimate their knowledge or underreport their attitudes and practices. Additionally, the study's focus on a specific region may limit the generalizability of the findings to broader, more diverse populations. Besides, the pandemic may have temporarily enhanced or diminished students' mental health literacy due to the heightened focus on mental health in the media and online platforms. This could affect the generalizability of our results to non‐pandemic conditions. Despite these limitations, the study's strengths lie in its large sample size and comprehensive analysis of the interplay between KAP regarding depression, providing valuable insights for developing targeted mental health interventions in university settings.

The study highlights a critical gap in the knowledge of depression among university students, despite a generally positive attitude and a tendency toward passive practice in managing this mental health issue. To effectively address the knowledge gap in depression among university students, it is crucial to innovate the format and teaching methods of mental health education programs and courses. These initiatives must be engaging, interactive, and tailored to the specific needs of diverse student demographics. Incorporating practical and motivational elements is key, such as interactive workshops, real‐life scenario simulations, and the use of digital platforms that resonate with a tech‐savvy generation. By focusing on practical skill‐building in areas like stress management and resilience, alongside providing a comprehensive understanding of mental health issues.

## AUTHOR CONTRIBUTIONS

**Xuechao Wang**: Conceptualization; methodology; writing—original draft; writing—review and editing. **Cuiluan Li**: Conceptualization; methodology; formal analysis; writing—review and editing; writing—original draft; data curation.

## COMPETING INTERESTS

The authors declare that they have no competing interests.

### PEER REVIEW

The peer review history for this article is available at https://publons.com/publon/10.1002/brb3.70030

## Supporting information



Supporting Information

## Data Availability

All data generated or analyzed during this study are included in this published article and its Supplementary Information files.
