# Supplementary material for: Knowledge, attitude, and practice of depression among university students
Source: Brain Behav. 2024 Sep 18;14(9):e70030. doi: 10.1002/brb3.70030 (PMC11410866; doi:10.1002/brb3.70030)
Supplement: Supplementary file 1 — Supporting Information [file BRB3-14-e70030-s001.docx]

|  | Coefficient of difficulty |
| --- | --- |
| **Knowledge** |  |
| 1. What are the main symptoms commonly associated with depression? (Multiple choices allowed) | 0.86 |
| 2. Depression is closely related to genetic factors, often seen in first-degree relatives of depressed patients, with a high concordance rate in monozygotic twins. | 0.48 |
| 3. What are the reasons that make college students prone to depression? (Multiple choices allowed) | 0.86 |
| 4. Women are more likely to suffer from depression than men. | 0.41 |
| 6. Which of the following diseases may contribute to depression? (Multiple choices allowed) | 0.73 |
| 7. Depression, in turn, can lead to greater stress and functional impairment, affecting the patient's life and exacerbating depressive symptoms. | 0.83 |
| 8. Patients with cardiovascular diseases, cancer, diabetes, and respiratory system diseases are more prone to depression. | 0.63 |
| 9. Depression can be treated with psychotherapy and medication. | 0.88 |
| **Attitude** |  |
| 1. If you observe symptoms of depression in friends or classmates, would you proactively help them? | 0.87 |
| 2. Are you willing to share your own emotions and psychological issues with others? | 0.76 |
| 3. Do you consider the role of mental health education on university campuses important? | 0.90 |
| 4. Do you believe you can get along well with others in your family, society, and school? | 0.84 |
| 5. Do you think you might suffer from depression? | 0.68 |
| 6. Regarding the occurrence of depression, do you believe you can acknowledge and face it, actively seeking help or finding ways to overcome it? | 0.84 |
| 7. Depression is something shameful or stigmatized. | 0.83 |
| **Practice** |  |
| 1. Is there frequent promotion or educational activities related to depression at your school? | 0.64 |
| 2. Do you often experience symptoms of depression? | 0.83 |
| 3. Do you often feel stress from family, school, society, etc.? | 0.68 |
| 4. Do you often use self-relief methods to vent negative emotions? | 0.75 |
| 5. In situations where self-relief of negative emotions is not ideal, do you actively seek help? | 0.63 |
| 7. Do you have alcohol dependence and often use alcohol to release emotions? | 0.93 |
| 8. Do you often feel physically uncomfortable? | 0.82 |
| 9. Do you feel anxious or nervous because of physical discomfort? | 0.79 |
| 10. Have you ever engaged in non-suicidal self-harm behaviors? | 0.95 |
